# Professional Atmosphere and Morals

**Published:** 1898-07

**Authors:** B. Holly Smith

**Affiliations:** Baltimore, Md.


### PROFESSIONAL ATMOSPHERE AND MORALS.[1]

BY B. HOLLY SMITH, M.D., D.D.S., BALTIMORE, MD.

THE American people are engaged to-day in a struggle undertaken at great sacrifice with a certain enormous expenditure of money and a probably appalling destruction of human life, yet undertaken with cheerful enthusiasm because it is in the interest of humanity.

It took many years of shocking barbarism at their very doors to force the United States to take this view of the situation, for we are a long-suffering people, patient to endure and hard to arouse. Besides, we are a very busy people. We have devoted our energies to the development of our material interests, and the accumulation of enormous fortunes with such immense success that no other interest has seemed to have any permanent attraction for us.

We have, in many and flagrant instances, suffered misgovernment and corruption, imposition and fraud, insolence and quackery, because we have been too busy to correct and punish them. Such is the interest displayed in the amassing of large fortunes, such the influence and respect of wealth, that to strangers' eyes we have appeared a sordid and materialistic people, absorbed in ignoble occupations, sunk in the greed of gain, too low to feel the aspirations of noble spirits, or to chivalrously resent insult and contumely, or to extend the helping hand of pity and love to an oppressed and downtrodden people. Foreigners, looking but at the surface, gazed with wonder at our enormous activity, seemed to see with surprise its only end, and summarized what they deemed our national characteristic in the contemptuous phrase, "A nation of shop-keepers."

Have we been to blame in this respect? Has not the impression which epitomized itself in these terms of contempt and low estimation some rational grounds for its making? And do we not need as a nation to regard wealth more as a means and less as an end? These are questions for statesmen and moralists, but their proper answer is brought home to every one of us in his peculiar vocation.

There is an excuse, and one which very nearly palliates and condones the offence, for the man or for the class of men whose lives have been devoted to the absorbing cares, the fierce competitions of business, to regard everything from a mercantile point of

---

[1] Read before The New York Institute of Stomatology.

view, to judge and decide all questions according to the standard of profit and loss.

We pardon the ferocity and cruelty of the soldier in the heat of the engagement, and we overlook the calculation and sordidness of the merchant in the fierce competition of trade. To the one all the kinder impulses are for the moment overwhelmed in the desire for conquest, in the other all humane considerations disappear in the lust for gain. We dismiss the barbarity of the first by saying, "This is war," and we overlook the inhumanity of the second by saying, "This is business."

But the question which concerns us more nearly at the present time is, To what extent have the so-called business principles penetrated what we have been accustomed to regard as the liberal callings, the professions, in American life? How seriously has the infection of mercantilism contaminated the professional atmosphere? For we hold that in the light of the professions' noble history, and by the lessons of those honored traditions which epitomize that history's teaching, and under the guidance of that spirit which informs and elevates professional life, the professional attitude and the mercantile attitude towards the world are entirely different and distinct. For the elemental and basic principle of mercantile life is annihilating competition; its successful and logical development is monopoly, and its successful and logical result is oppression.

One of the greatest misfortunes of mercantile occupations is that the man with the lowest moral tone, devoid of benevolent impulses and lacking humanitarian ideas, is able to set the standard for the entire class. For he who cheapens production, and in cheapening production, as one factor of the operation, lowers the standard of living among his employees, can undersell his more humane and benevolent rival, who must either follow the pernicious example or suffer a crushing mercantile defeat from which there is no revival.

The history of great mercantile successes is replete with incidents to verify this statement, and in all important details could have been foretold from a knowledge of the principles upon which these successes are based.

The fact that suffering and oppression are not the aim, but the unregarded incidents of these successes, mitigates in no degree the cold and cynical selfishness of the mercantile atmosphere. This is necessarily an atmosphere of selfishness, and neither logic nor common sense permits us to apply the terms selfish and unselfish to the same class of actions. Nor do the many examples of philanthropy performed by those who have acquired wealth confute the fact that these hard, unfeeling, inhuman conditions necessarily obtain in the practice of that wealth's acquisition.

We scorn to impute to these philanthropists either concession to outraged consciences or unworthy desires for enduring monuments, and we will credit them with having become wealthy in honorable business ways, and yet we will assert that the controlling principle of business life and methods is intelligent and calculating selfishness.

The question that naturally arises is, Do we of the liberal professions claim no higher motive? If we do not, then I do not conceive that we can do better than to adopt and practise these twentieth century shop-keepers' methods.

In that event our talents, our acquired abilities, our inherited learning, the wisdom which generations of self-sacrificing predecessors have placed at our disposal, are but factors and elements in the great task of personal aggrandizement, and must be used as means of our personal profit. Above all, our reputations are salable articles, to be purchased by the highest bidder for the greatest cash consideration.

Any additions which may be made to the large knowledge or more facile practice of our profession are primarily ours, and incidentally the profession's, provided they pay us well for it, and as for humanity,—suffering humanity,—the same course is open to them, and business rivalry may do much for them by cheapening the inventions of competing inventors.

In this cheerful view of our rights and privileges, the title of honor is but the right of advertising, the diploma, the license to compete with other tradesmen in specific merchandise, and it is but a short step to drummers or travelling salesmen.

Traditions of so-called honorable and established practice are proscriptive and restrictive incidents of limited business areas and old-fogy methods, to be eliminated in this progressive age. But are we ready to go into business on these terms or with these methods? Are we prepared to relinquish a professional standing, and to appeal to the world as a business organization? Do we believe that the world holds the liberal professions less in honor or esteem to-day than before? If this be true, and I for one am prepared most fervently to doubt and to deny it, where does the fault lie? With us or with the world?

Of one thing we must be assured, the world cannot take us more seriously than we take ourselves; it will not rate us above our own estimate. If what we assume as our profession is a purchasable commodity, it will buy it as such and will not once resent the fact that we cheapen ourselves.

Gentlemen who resent the introduction of what they call sentiment into the practical affairs of our professional life have several things to consider, possibly some things to learn.

It is unfair, if the traditions of dentistry *are* unsettled, to sweep aside, unargued and unanswered, the conscientious convictions of thoughtful and earnest men, provided we are to adopt, although it be but tacitly, a body of principles upon which our conduct as a profession is based.

It is ungenerous to dismiss considerations which, though they may discriminate against the individual, are, nevertheless, for the general good.

It is unwise to refuse to weigh carefully the advantages and disadvantages of any line of action, however seductive the profitable results which it promises may appear, or to decide questions fraught with grave consequences upon the semblance of a present profit.

We have been accustomed in the past to associate the words *honor* and *dignity* with professional life, and to attach to them when so associated a peculiar significance. We have certainly implied their presence and their union in the term *professional*, and have branded their absence or their disgrace by the term *unprofessional.*

If these terms retain the sacred meaning hitherto accepted and understood by all, I submit that it would be the height of folly for dentistry to dismiss them from its vocabulary, or to connive at actions within the profession to which they cannot be fully and truly applied. If they have lost their significance, then, in Heaven's name, let us adopt some words which mean what they used to mean.

Do we need here, and at this time, to state again for the benefit of our practical friends our firm and abiding conviction that dentistry is a profession and a liberal profession? I sincerely believe that the most cynical among them will spare us this humiliation, that they must, if from no other cause, from very shame concede us that vantage point. If they do not, we can have no common ground of debate, for it is beneath my poor power to strengthen the arguments already advanced on that point, and I shall not attempt it.

But the objection may be urged that the liberal training, the exceptional skill, and complete devotion necessary to make a dentist, while they may establish dentistry as a profession, are not to be urged as arguments against the dentist's right to use as his own private property his ability, his talents, his reputation, the results of that training, skill, and devotion.

It is in opposition to this position, stated, I think, with perfect fairness, that I desire as a dentist to utter my most respectful and emphatic protest. I crave my brother's forgiveness if this is any attack upon his personal rights, but I justify my protest on the ground that it is to his action as a professional man, and not as an individual, that I take exception. And even with that justification I would hesitate did I not feel convinced that the weight of wisdom and experience in the profession approve. For I believe, though we may frequently lose sight of the fact, that our sober thought must convince us that every liberal profession has a solemn duty and responsibility, first to humanity, and second to its own membership.

This is no new creed, but is the strongly stated conviction of the sages and philosophers of generations. To its true believers has been given the supreme glory of extending the empire of knowledge, truth, and happiness. Their sacrifices of life, fortune, and talent have ripened for us into a harvest of knowledge, skill, and power, and their spirits cry out against the abuse of this sacred inheritance.

As one of the first successful specializations of the great medical and surgical art, medicine, the attitude of dentistry towards humanity is that of a healer—a benefactor. Its mission is to alleviate suffering and to increase the aggregate of human happiness by reducing the sum of human misery. Based on scientific knowledge, through physical means, it deals with vital forces, and helps to supply the healthy body without which the moral and spiritual tone of individuals and communities is inevitably lowered. Its practice has in view the conservation of vitality, the preservation of health, the prolongation of life.

The duty of dentists to the profession is the logical corollary of the profession's duty to humanity. To fellow-members of the same learned and liberal brotherhood, do we not owe mutual helpfulness, that the effectiveness of our profession may be extended and increased?

For the same and for the good of the profession, that the body of its knowledge be widened, that the skill of its practice be improved, that the sum of its benefactions be enlarged, does not a liberal and humane policy dictate a brotherly sharing of the results of individual investigation?

It appears to me that such must be our conclusion, if we realize and appreciate the responsibility of our duty to mankind and to one another. And it certainly seems to me that all reputable and honorable traditions of medicines ought to obtain in their fullest force and effect in dentistry.

This much for convictions which have become fixed with some firmness in my own mind, and which I hope and believe are shared by many of my professional brethren.

What is the practical effect upon us as a profession, and how does it react upon individuals if we ignore a professional standard of conduct and set up the mercantile motto *laissez faire* as our guide and rule? To some favored individuals the result would be temporarily advantageous, no doubt, but to the profession at large, and eventually to the individual members, it would be a distinct disadvantage. We think we can give a proof of this proposition sufficiently convincing to the fair-minded to turn the chief arguments relied on by the opposition directly against them.

We believe it can be safely asserted that just as soon as personal advantage is set up as the supreme standard of human conduct, the taint of insincerity will be imputed, as affecting judgment and consequent action, discrediting and cheapening both. In other words, if you are for sale, and I know you are for sale to the highest bidder, and you deal in an intangible something of which I am not a judge, how do I know you are not selling something you do not possess, a knowledge you do not have, a skill of which you are not master, simply because the assertion that you do have them will bring you a price? When you have brought the public to that point, you have cheapened and discredited all assertions of professional skill and knowledge, provided such assertions are sold.

An actress or a divine says, over her or his signature, that a certain soap possesses superb cleansing qualities. This, well put forward in illustrations and placards, is a good advertisement for the soap, but not for the actress or the divine or for their professions.

A man with fierce moustache and haughty military bearing stalks between the placards bearing the announcement, "I use Get-there's Sovereign Panacea." We may be forced to note the name of the medicine, but how do we regard the unfortunate man?—or the class of men thus honestly employed? And what professional reputation in the world could stand the shock of the humiliation and degradation of such an attitude?

Is it not as clear as the noon-day sun that such a sale of a professional opinion, while it may doubtless be excellent advertising, humiliates, belittles, and degrades the professional reputation, whether of the individual or the class?

While the world wags, we must judge the action by the motive.

The humblest speaker who delivers his message from the heartfelt sense of duty carries more weight than the most learned and flowery orator preaching for pay.

I am aware that the contention that an unprofessional use of professional skill, ability, or reputation is impolitic, and should for that reason be abandoned, is open to serious criticism. It abandons the right or wrong of the question and appeals to policy. But I use this argument of disadvantage simply to offset the argument of personal advantage, not as the ground of my opposition.

Call me sentimentalist, idealist, theorist, or what you may, I am content to rest my claims for a strictly professional use of professional advantages upon time-honored traditions, founded upon the discrimination which generations of thinkers have established between the professional and the mercantile attitude towards mankind and towards each other.

If this position be narrow and unprogressive, I nevertheless rejoice in it, for I believe it is right.

